# Supplementary material for: Modeling glioblastoma heterogeneity as a dynamic network of cell states
Source: Mol Syst Biol. 2021 Sep 16;17(9):e10105. doi: 10.15252/msb.202010105 (PMC8444284; doi:10.15252/msb.202010105)
Supplement: Supplementary file 6 — Source Data for Figure 5 [file MSB-17-e10105-s004.zip › Figure5A_sourcedata/GSEA_3071/hallmarks_stateVI.GseaPreranked.1623742557031/HALLMARK_HEDGEHOG_SIGNALING.html]

Details for gene set HALLMARK\_HEDGEHOG\_SIGNALING[GSEA]

|  || Dataset | state63071 |
| Phenotype | NoPhenotypeAvailable |
| Upregulated in class | na\_neg |
| GeneSet | HALLMARK\_HEDGEHOG\_SIGNALING |
| Enrichment Score (ES) | -0.45635596 |
| Normalized Enrichment Score (NES) | -1.545787 |
| Nominal p-value | 0.064425774 |
| FDR q-value | 0.15496293 |
| FWER p-Value | 0.832 |
Table: GSEA Results Summary

  

Fig 1: Enrichment plot: HALLMARK\_HEDGEHOG\_SIGNALING      
 Profile of the Running ES Score & Positions of GeneSet Members on the Rank Ordered List

  

| PROBE | GENE SYMBOL | GENE\_TITLE | RANK IN GENE LIST | RANK METRIC SCORE | RUNNING ES | CORE ENRICHMENT || 1 | ETS2 |  |  | 52 | 0.864 | 0.0563 | No |
| 2 | MYH9 |  |  | 312 | 0.534 | 0.0285 | No |
| 3 | GLI1 |  |  | 786 | 0.379 | -0.0710 | No |
| 4 | PML |  |  | 874 | 0.365 | -0.0652 | No |
| 5 | L1CAM |  |  | 971 | 0.350 | -0.0631 | No |
| 6 | HEY2 |  |  | 2101 | -0.297 | -0.3506 | No |
| 7 | VEGFA |  |  | 2104 | -0.298 | -0.3268 | No |
| 8 | THY1 |  |  | 2265 | -0.326 | -0.3443 | No |
| 9 | UNC5C |  |  | 2672 | -0.417 | -0.4223 | Yes |
| 10 | CNTFR |  |  | 2748 | -0.440 | -0.4070 | Yes |
| 11 | RTN1 |  |  | 2785 | -0.451 | -0.3800 | Yes |
| 12 | TLE1 |  |  | 2817 | -0.462 | -0.3508 | Yes |
| 13 | CRMP1 |  |  | 3039 | -0.558 | -0.3662 | Yes |
| 14 | CDK6 |  |  | 3234 | -0.704 | -0.3622 | Yes |
| 15 | DPYSL2 |  |  | 3544 | -1.298 | -0.3414 | Yes |
| 16 | NRCAM |  |  | 3614 | -2.198 | -0.1806 | Yes |
| 17 | NRP2 |  |  | 3617 | -2.285 | 0.0058 | Yes |
Table: GSEA details [plain text format]

  

Fig 2: HALLMARK\_HEDGEHOG\_SIGNALING: Random ES distribution      
 Gene set null distribution of ES for **HALLMARK\_HEDGEHOG\_SIGNALING**
